# Validating a modified instrument for measuring Demand-Control-Support among students at a large university in southern Sweden

**DOI:** 10.1080/16549716.2023.2226913

**Published:** 2023-06-26

**Authors:** Jack W. Palmieri, Anette Agardh, Per-Olof Östergren

**Affiliations:** Social Medicine and Global Health, Department of Clinical Sciences, Lund University, Malmö, Sweden

**Keywords:** University students, Demand-Control-Support, study environment, factor analysis, sexual harassment

## Abstract

**Background:**

University students experience a distinct working environment in the context of completing their studies. In line with existing research into the connection between workplace environment and stress, it is rational to believe that such study environments can affect the level of stress that students experience. However, few instruments have been developed for measuring this.

**Objective:**

The aim of this study was to validate a modified instrument based on the Demand-Control-Support (DCS) model among students at a large university in southern Sweden to determine its utility for assessing the psychosocial properties of the study environment.

**Methods:**

Data from a survey performed at a Swedish university in 2019, which generated 8960 valid cases, was used. Of these cases, 5410 studied a course or programme at bachelor level, 3170 a course or programme at master level, and 366 a combination of courses and programmes on the two levels (14 missing). A 22-item DCS-instrument for students was used comprising four scales: Psychological workload (demand) with nine items, Decision latitude (control) with eight items, supervisor/lecturer support with four items, and colleague/student support with three items. Construct validity was examined using exploratory factor analysis (EFA) and internal consistency using Cronbach’s alpha.

**Results:**

The results of the exploratory factor analysis of the Demand-Control components support a 3-dimension solution with dimensions corresponding to psychological demands, skill discretion, and decision authority in the original DCS model. Cronbach’s alpha coefficients were acceptable for Control (0.60) and Student Support (0.72) and very good for the Demand and Supervisor Support scales (0.81 and 0.84, respectively).

**Conclusions:**

The results suggest that the validated 22-item DCS-instrument is a reliable and valid tool for assessing Demand, Control, and Support elements of the psychosocial study environment among student populations. Further research is necessary to examine the predictive validity of this modified instrument.

## Introduction

Universities can be understood as workplaces for students, as well as complex sites for study and social interaction. Students can therefore have specific expectations on psychosocial workplace environment and protection. In this context, psychosocial workplace can be understood as the combination of social and psychological job characteristics [1]. It is rational to believe that such study environments can affect the level of stress that students experience, and existing research has shown that stress is a common occurrence among this population and associated with a number of negative outcomes for the students’ academic performance [2]. Before actions can be taken to better address these high levels of stress, appropriate tools must exist to measure the psychosocial conditions of the study environment. Despite this, few instruments have been developed and validated for measuring this phenomenon.

In traditional workplaces, the Job-Demand-Control model (or job strain model) is one of the most influential and widely used models of occupational stress, appearing in numerous publications examining connections to, among other outcomes, psychological wellbeing [3], cardio-vascular disease and blood pressure [4], and musculoskeletal disorders [5]. First developed by Karasek in 1979 utilising data from Sweden and the USA, the model postulates that work stress primarily comes from the interaction between psychological demands due to work and the effect of lack of decision latitude that allows employees to make their own decisions and enhance job satisfaction, commonly labelled ‘control’ [6]. In 1988, the model was expanded to acknowledge the importance of social support as a potential buffer or, in its absence, as an additional stressor [7]. This expanded model will be referred to as the Demand-Control-Support model (DCS) in this article.

The DCS model has been examined in both cross-sectional and longitudinal research [3]. Results of these studies have been mixed, supporting both the additive [3] and buffer hypotheses [8]. In a large systematic review and meta-analysis of work environment and depressive symptoms research, Theorell et al. concluded that there is ‘substantial empirical evidence between lack of decision latitude, job-strain, and bullying and depressive symptoms’ [9]. The findings indicated moderate evidence (the highest possible level for research in this study according to the GRADE criteria [10] as no randomised trials were included) for control/decision latitude as protective factors against depressive symptoms, as well as for job strain (high demands and low control) as a harmful factor associated with depressive symptoms. Limited evidence was found for the relationship between psychological demands, passive jobs (low control, low demands), high-pressure jobs, and low support at the workplace, in relation to depressive symptoms [9].

The DCS model is often operationalised by the Job Content Questionnaire (JCQ) and the shorter Demand Control Support questionnaire (DCSQ), both self-administered instruments.

The JCQ has been validated in, among other languages, Swedish, Norwegian, German, and English [11–13]. The JCQ and the shorter DCSQ are general instruments used across a variety of different occupations including white collar employees of different groups [13], teachers [14], firefighters [15], nurses and other health care workers [16] to examine psychosocial work environment and associated outcomes in stress, psychological health, and burnout. Although sometimes used for specific work categories, the strength of the JCQ instrument is its ability to be applied across occupations and heterogenous populations [1].

Less research has been conducted utilising the Demand-Control-Support (DCS) model to examine the psychosocial study environment of university students’ ‘workplace’. Existing studies focus on internships and work placements that resemble traditional workplaces [17] or are smaller experimental studies that do not address the study environment as a whole [18]. Previous studies share a common result: a negative correlation between decision latitude and perceived demands, which is unexpected when compared to the original model assumptions [18,19]. Where studies have used the DCS model in student settings, they use a variety of different scales drawn from different instruments [20]. In some studies this includes using a version of the JCQ (23 items) that may have ‘left out some specific features related to the academic context’ as no students were involved in defining the most important areas [21] or instruments based on the DCS model but with only 2–3 items per variable [19].

Two studies have gone further in adapting versions of the JCQ for the student context. In a study by Schéle and colleagues among 322 dental students undergoing clinical training at four universities in Sweden [22], a version of the JCQ Adapted for Dentistry students was used to examine environmental and individual characteristics related to stress. The study concluded that the psychosocial work environment of the students included in the study produced high levels of perceived stress [23]. This instrument had been adapted for a specific set of students and had not been validated. A study conducted among 146 psychology students at two universities in Germany also utilised an adapted version of the JCQ [24]. This version had been pretested among students and internal consistency had been measured, but no factor analysis was conducted.

In 2018, Lund University initiated the Tellus Project concerning sexual harassment among students and employees of the university. The project used a cross-sectional study design and an online questionnaire for data collection. This questionnaire included questions about study environment demand, control and support. The instrument used is a version of the instrument developed by Schéle and colleagues [22] that had been modified by researchers at Lund University in collaboration with students and student organisations. In this article, this instrument is named the DCS-instrument.

The aim of the current study was to examine the construct validity and internal consistency of the Demand-Control-Support (DCS)-instrument among students at Lund University, Sweden, to determine whether it is an appropriate tool for measuring the psychosocial study environment in this setting.

## Methods

### Study design and data collection

A population-based cross-sectional study was conducted among students at Lund University as part of the Tellus project. The Tellus project is a three-year, research-based project concerning sexual harassment among students and employees at Lund University, a public university in southern Sweden with eight faculties and approximately 40,000 students.

All undergraduate and graduate students registered for studies during the autumn term of 2019 were invited to participate in an online self-administered questionnaire via their registered email addresses. The email text contained information about the study and contact details for those responsible. It also contained a link to the web-based questionnaire available in Swedish and English. Prior to answering the questionnaire, participants were asked to provide consent.

The survey instrument was developed based on a literature search and information gathered through a series of seven focus group discussions and 20 individual interviews with students at the university. The instrument had 117 questions divided into eight sections. These sections included sexual harassment, study environment, health, trust, and confidence as well as experiences of other types of harassment and derogatory treatment. Ethical approval was received from the Swedish Ethical Review Authority (number 2018/350).

### Study measures

#### Demand-Control-Support instrument

The instrument used in this study is a modified version of the Job Content Questionnaire (JCQ) that was adapted for assessing dentistry students’ study environment by Swedish researchers [22]. This instrument is named the DCS-instrument.

As our study included students from all faculties of Lund University, the instrument adapted by Schéle and colleagues was deemed to be too narrow in its formulation. Therefore, the authors tested the questions with students and representatives of student organisations to optimise question interpretation. Priority was given to the original formulation in the JCQ wherever differences occurred.

**Figure 1.**
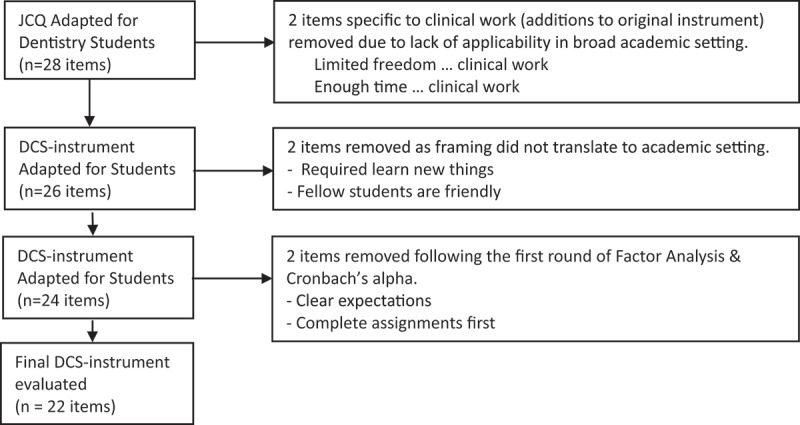
Flowchart of changes made to the instrument during the study.

**Figure 2.**
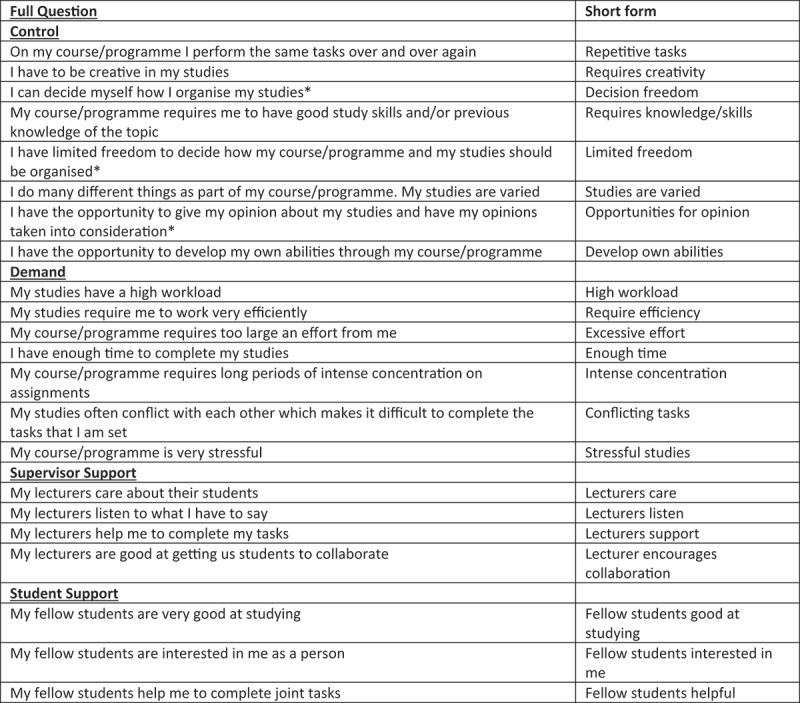
Modified 22-item Demand-Control-Support instrument (English version) for measuring psychosocial study environment and shortened question forms used in this article.

Based on this ‘face validity’ testing, some modifications were made. First, the instrument was shortened to maintain the balance among decision authority, skill discretion, psychological demands, and supervisor and co-worker support found in the original scale. Based on feedback from the students, two items were removed from the scales, one from psychological demands (My studies require me to learn new things) as this was taken to be a general expectation in a university setting, and one from student support (My fellow students are friendly) as there was a lack of consensus and conceptual clarity on this question. Other questions were reworded to bring them in line with the academic environment and student interpretation. A flowchart detailing this process is found in Figure 1. The final DCS-instrument tested in this paper comprised nine items to measure demands, eight items for control, four items for supervisor/lecturer support, and three items for colleague/student support. Each item was answered on a nominal Likert-like 4-point scale. The final modified version of the instrument is shown in Figure 2.

#### Background variables

*In this study, Gender Identity* was assessed with two questions; ‘What gender were you assigned at birth’, and ‘What is your current gender identity’. The second question had three options: female, male, and I do not identify as female or male. The answer to ‘current gender identity’ was used where provided, with ‘gender assigned at birth’ used for those cases without an answer for current gender identity. Respondent’s *Age* was recorded as ‘18–25’, ‘26–30’, ‘31–40’ and ‘41 years or older’, and their *Country of birth* was assessed as ‘Sweden’, ‘In a Nordic Country (not Sweden)’, ‘Europe (not a Nordic country)’ or ‘Outside of Europe’. The number of Semesters studied at Lund University by the respondent was recorded using the following question ‘How many semesters have you studied at Lund University in total? (Including the current semester)’, with options ‘0–1’, ‘2–3’, ‘4–5’, ‘6–7’, ‘8–9’, ’10–11’ and ‘More than 11’.

#### Statistical analysis

Two parameters for validity were tested in this study: construct validity and internal consistency.

Construct validity was calculated to measure dimensionality and the extent to which the sub-scales of the construct in question were measured. With reference to the underlying constructs of the DCS model, and an appreciation that significant changes had been made to the instrument warranting a more unrestricted analysis, an exploratory factor analysis (EFA) using principal factor extraction and varimax rotation was selected. Internal consistency was evaluated using Cronbach’s alpha and item-total correlations to measure reliability. Statistical analysis was conducted using Stata 16 [25].

## Results

### Socio-demographic characteristics

A total response rate of 32% was achieved. Respondents with data missing for sex and gender (*N* = 69), age (*N* = 46), questions on sexual harassment (*N* = 74), or on one or more items of the DCS-instrument (*N* = 707), were excluded from the analyses. Data collection was part of a broader project on sexual harassment at Lund University, and the questionnaire was developed with this focus. In this context, having answered these questions was seen as important for inclusion in the data set. This resulted in a study population of 8960 respondents. A simple non-response analysis showed strong similarities between the study participants and the total population in a number of key characteristics [26]. Characteristics of the study sample are provided in Table 1.

**Table 1.** Prevalence of socio-demographic factors among study sample of Lund University students who had responded to all items in the 22-item DCS-instrument (*N* = 8960).

	All		Female		Male		Neither female nor male	
Variables	n	%	n	%	n	%	n	%
**Sex**								
Female	5596	62.5						
Male	3301	36.8						
Neither female nor male	63	0.7						
**Age**								
18–25	6966	77.8	4347	77.7	2574	78.0	45	71.4
26–30	1184	13.2	732	13.1	443	13.4	9	14.3
31–40	509	5.7	315	5.6	187	5.7	7	11.1
>41	301	3.4	202	3.6	97	2.9	2	3.2
**Study level**								
Bachelor	5410	60	3563	64	1806	55	41	65
Master	3170	35	1824	33	1329	40	17	27
Combination	366	4	199	4	162	5	5	8
*(Missing)*	*(14)*		(10)		(4)		*(0)*	
**Country of birth**								
Sweden	7161	79.9	4446	79.5	2673	81.0	42	66.7
Nordic (not Sweden)	209	2.3	147	2.6	59	1.8	3	4.8
Europe (not Nordic)	769	8.6	496	8.9	261	7.9	12	19.1
Outside Europe	816	9.1	504	9.0	306	9.3	6	9.5
*(Missing)*	*(5)*		*(3)*		*(2)*		*(0)*	
**Semesters studied**								
0–1	2721	30.4	1734	31.0	968	29.3	19	30.2
2–3	2191	24.5	1456	26.0	715	21.7	20	31.8
4–5	1559	17.4	964	17.2	590	17.9	5	7.9
6–7	1113	12.4	704	12.6	401	12.2	8	12.7
8–9	758	8.5	430	7.7	322	9.8	6	9.5
10–11	391	4.4	208	3.7	182	5.5	1	1.6
>11	224	2.5	100	1.8	120	3.6	4	6.4
*(Missing)*	*(3)*		*(0)*		*(3)*		*(0)*	

#### Construct Validity

Prior to conducting the exploratory factor analysis, a series of methodological decisions were made following the steps outlined by Watkins [27], to ensure the correct application of methods and increase interpretability.

Various authors recommend including a minimum of 3–6 variables for each common factor [28]. With eight and nine variables for demand and control respectively, all items were included in the initial analysis. When considering sample size, allowance should be made for the absolute number of participants, as well as the ratio of participants to measured variables [27]. Following Comrey and Lee [28], an absolute number of 1000 and a ratio of 20:1 or higher are considered excellent. The current study had 8960 participants and a ratio of 373:1. As EFA assumes an underlying normal distribution, univariate skew and kurtosis were measured. A normal distribution will have a skew = 0 and kurtosis = 0, and in this data set the range was low (Range −0.70–0.71 and −1.1–0.1, respectively). Using the ‘standard’ values of skew >2 and Kurtosis >7 indicating univariate non-normality [29], the results suggest that this data exhibits acceptable univariate normality. Multivariate normality was tested with Mardia’s skew. The test produced multivariate skewness of 5.69, suggesting some departure from normality (Reference 0).

Bartlett’s test of sphericity and the Kaiser-Meyer-Olkin measure of sampling adequacy were used to ascertain whether the dataset was appropriate for data reduction techniques such as EFA. As the p-value of Bartlett’s test of sphericity (*p* < 0.001) is below the 0.05 significance level utilised in this paper, this dataset is considered suitable for data reduction techniques such as EFA [30]. In addition, the Kaiser-Meyer-Olkin Measure of sampling adequacy (0.851) was above the commonly recommended value of 0.7 [31] and thus factor analysis was considered suitable for these items. Due to the presence of (low level) multivariate nonnormality (as measured by Mardia’s Kurtosis of 341.73 compared to a reference of 323), Principal Factor Extraction was chosen as the extraction method [32]. This is supported by findings in previous research for example de Winter and Dodou, 2011 [33].

Although several methods exist to guide selection of the number of factors to retain in the analysis, no ideal method has been identified [34]. Thus, Parallel analysis (four factors), Minimum Average Partial Correlation (four factors) and a scree plot examination were conducted (four factors) to aid in the selection of the number of factors to retain [34]. Based on these findings and the theoretical assumptions of the demand-control model, 2 and 3 factor models were selected for theoretical interoperability and best fit. Previous studies have shown that Control can sometimes be separated into skill discretion and decision authority, the foundation of the 3-factor model.

Both varimax (orthogonal) and Promax (oblique) rotations were tested, and the results were compared. Varimax was selected due to its commonness in similar research [35], and Promax due to the fact that it is an oblique modification to the varimax procedure [36]. Based on the similarities of the results, the theoretical assumption of a lack of correlation between the factors, and to improve the ease of interpretation [37], the decision was made to apply varimax rotation.

An initial EFA using principal factor extraction and varimax rotation was conducted for the 17 Demand and Control items in 2 and 3 factor models consecutively. Factors with loading of 0.3 or higher were considered to load adequately with moderate correlation [38].

Results from this analysis with the two-factor model grouped items connected with ‘Control’ under factor 2 except for ‘Requires skills that showed adequate factor loading (0.6) on factor 1. Factor 1 grouped items connected with ‘Demand’ except for ‘Clear expectations’ and ‘Complete assignments first’. ‘Clear expectations’ had adequate loading (0.5) on factor 2, and ‘Complete assignments first’ did not have adequate factor loading on either factor.

The 3-factor solution showed a clear meaning for the third factor within the ‘Decision Authority’ area of ‘Control’. Two of the three items showed strong loading, while the third ‘Lots to say’ did not load on this factor. As with the two-factor model, the items grouped under factor 2 connected strongly with skill discretion except for ‘Requires skills’ that loaded onto factor 1 (Factor loading 0.6). Factor 1 contained two items, ‘Clear expectations’ and ‘Complete assignments first’, that continued to load onto other factors or showed insufficient loading.

### Removal of two items

After reviewing the preliminary results of the factor analysis and Cronbach’s alpha, items 13 (‘The expectations my education places on me are clear regardless of where the expectations come from’) and 17 (‘I often complete my assignments before my classmates’) were removed. Despite some items with low Cronbach’s alpha, this analysis and the Factor Analysis combined led to the decision to retain all other items. Only the 3-factor model was retained, shown in Table 2.

**Table 2.** Exploratory factor analysis using varimax rotation for 15-items of the DCS-instrument adapted for study environment at Lund University (*n* = 8960). Three factor solutions shown. Only factors with loading > 0.3 are shown.

Dimension	Item		Factor Loading		
			Factor 1	Factor 2	Factor 3
Control	Repetitive tasks§			0.54	
	Requires creativity			0.52	
	**Decision freedom**				**0.81**
	Requires knowledge/skills		0.61	(0.21)	
	**Limited freedom§**				**0.65**
	Variety in studies			0.78	
	**Opportunities for opinion**			**0.58**	(0.25)
	Develop own abilities			0.67	
Demand	High workload		0.84		
	Require efficiency		0.78		
	Excessive effort		0.66		
	Enough time§		0.52		
	Intense concentration		0.75		
	Conflicting tasks		0.55		
	Stressful studies		0.77		

Bold text – items on decision authority sub-scale.

§ - Item score reversed.

The results of the revised scales show modest improvements in the factor loading of factor 3 when compared to the results of the instrument with 17-items. The item ‘requires knowledge/skills’ loaded onto factor 1 as opposed to the expected factor 2, while Opportunities for opinion’ showed loading on factor 2 instead of the expected loading on factor 3. All items in the Demand scale showed adequate loading on the expected factor.

### Internal consistency

Cronbach’s alpha coefficients were calculated to map internal consistency for the three scales (Demand, Control, and Support). Internal consistency describes the extent to which all test items measure the same concept or construct and is based on the inter-relatedness of the items within the test [39]. Item-test correlation shows the extent to which each item is correlated with the overall scale, and the item-rest correlation shows how correlated an item is with the scale computed from all other items. Table 3 shows the results of this analysis.

**Table 3.** Item-test and item rest correlations and Cronbach’s alpha coefficients for items in the modified 22-item DCS-instrument among students at Lund University (*N* = 8960).

Item	Item-test	Item-rest	Alpha
**Control**			**0.60**
Repetitive tasks	0.42	0.21	0.60
Requires creativity	0.54	0.32	0.57
Decision freedom	0.52	0.30	0.57
Requires knowledge/skills	0.38	0.15	0.62
Limited freedom	0.40	0.16	0.61
Studies are varied	0.63	0.44	0.53
Opportunities for opinion	0.61	0.40	0.54
Develop own abilities	0.65	0.49	0.52
**Psychological Demands**			**0.81**
High workload	0.78	0.69	0.76
Require efficiency	0.68	0.57	0.78
Excessive effort	0.70	0.57	0.78
Enough time	0.66	0.53	0.79
Intense concentration	0.68	0.56	0.79
Conflicting tasks	0.64	0.50	0.79
Stressful studies	0.81	0.72	0.76
**Support from supervisor**			**0.84**
Lecturers care	0.85	0.72	0.78
Lecturers listen	0.85	0.73	0.78
Lecturers support	0.83	0.68	0.80
Lecturer encourages collaboration	0.78	0.60	0.84
**Support from fellow students**			**0.72**
Fellow students good at studying	0.74	0.46	0.71
Fellow students interested in me	0.82	0.54	0.63
Fellow students helpful	0.84	0.62	0.53

Item-test, item-rest and Cronbach’s alpha coefficients for the 22-item DCS-instrument are given in Table 3. Values for item-rest correlations for the psychological demand scale were all above 0.5 (range 0.50–0.72). This indicates a good correlation with the other items comprising the overall scale score when compared to the rule of thumb that item-rest correlations should be 0.4 or higher [34]. Items in the ‘Control’ scale showed weaker correlation, while the scales for supervisor and student support showed high correlation (ranges 0.46–0.62 and 0.60–0.73 respectively).

Following the general rule that a Cronbach’s alpha of 0.6–0.7 is acceptable and 0.8 is very good [40], the Cronbach’s alpha coefficients were acceptable for control (0.60) and student support (0.72) and very good for demand (0.81) and supervisor support (0.84).

## Discussion

The purpose of this study was to validate the 22-item DCS-instrument through construct validity and internal consistency to examine whether it could be used to assess the psychosocial study environment of university students. To our knowledge, no validated instrument exists to assess strain related to demand, control, and support in university students’ study environment. Since the Demand-Control-Support model is a well-established international theory utilised in many studies, validation of a version for university students would allow comparisons across universities and countries.

After removing two variables from the DCS-instrument used in the Tellus survey, resulting in the 22-item version, the results of the exploratory factor analysis support a 3-dimension solution to the instrument with dimensions corresponding to the psychological demands, skill discretion, and decision authority aspects of the original instrument, although the decision authority solution was supported by only two items. All items in the psychological demand scale loaded adequately onto the same factor, while one item each in the skill discretion and decision authority sub-scales showed insufficient loading on the expected factor. Previous research has shown higher correlations between skill discretion and decision authority, and more homogeneity in these concepts [41]. Having items that load between these two factors, as is the case with ‘Opportunities for opinion’, is therefore in line with the theoretical underpinnings of this model.

Cronbach’s alpha coefficients were acceptable for Control (0.60) and Student support (0.72) and very good for the Demand and Supervisor support scales (0.81 and 0.84, respectively). The results of the validation suggest that the modified 22-item instrument is reliable and valid for assessing Demand, Control, and Support dimensions of the study environment in student populations at universities in Sweden.

Two additional items could have been removed: ‘requires knowledge/skills’ and ‘opportunities for opinion’, as these did not adequately load onto the expected factors. Considering the combination of minimal advantages regarding Cronbach’s alpha to their removal and the tradition of maintaining the instrument as closely as possible to the original JCQ instrument to allow for comparisons, the decision was made to retain these.

Few comparable instruments for measuring Demand-Control-Support have been used to examine psychosocial study characteristics among university students. In one study among dental students in Sweden, Schéle et al. utilised a modified version of the JCQ instrument, and that is the basis for the instrument used in this study [22]. Although this instrument was not validated, in later research results were favourably compared with other scales measuring environmental stress [23]. Schmidt and co-authors conducted research at two German universities about Demand-Control, stress, and neuroticism using an adapted variant of the JCQ [24]. This instrument was pre-tested with a group of students, and internal consistency was established with Cronbach’s alpha scores. In their study, Control and Demand dimensions had alpha scores of 0.80 and 0.77 respectively, similar results for Psychological demands, but substantially higher scores for Control [24]. No evidence could be found of construct validity having been tested.

Existing research has been conducted using either general instruments designed for traditional workplaces [17] or modified instruments designed for a relatively narrow group of students (Dentistry students during their clinical work [22] and psychology students [24]). This study validates a more universally applicable instrument that has removed specialised items related to clinical work compared to the instrument used for dentistry students [22].

### Strengths and limitations

The large sample from a public university, which covers the full range of university studies, makes it easier to claim generalisable results as a strength of the study, as well as the implementation of the data collection in close cooperation with student representatives and the university management. The large sample also made it possible to apply the evaluation tools in a robust manner. Four key limitations have been identified in this study.

The first limitation is the possibility of selection bias. This can take the form of self-selection, whereby individuals select themselves for the survey on the basis of factors correlated with the measures of interest that in turn bias the results [42]. As this instrument was part of a questionnaire on experiences of sexual harassment, it is possible that those with experiences of sexual harassment could be overrepresented, especially considering the timing of the survey soon after the #metoo movement. However, with regard to questions on study-related stress, there is little reason to believe that this would have a systematic effect on the results.

The second limitation relates to the inability to differentiate between responses to the English vs. the Swedish version of the survey. As a new instrument in both languages, there could be differences in how the questions were interpreted. The translation was carried out by a native speaker, and back translation was utilised to examine differences in interpretation to minimise this. Despite this, there could still be differences in how respondents interpret the items in the two versions.

The third limitation is related to the relatively low Cronbach’s alpha coefficient for the control scale. Although this figure falls within the acceptable range [40], it is relatively low compared to the other scales and thus the internal consistency could be questioned.

The fourth limitation is the low number of items that load onto factor three (i.e. the decision authority dimension). Although two items can sometimes be used to identify a factor [27], three are generally needed for statistical identification [35].

Having validated this instrument among a student population will allow future research into the study environment and its associations with other outcomes to be conducted, and comparisons made across settings. One area for future research would be an examination of the predictive validity of this DCS-instrument.

## Conclusions

In conclusion, the findings of this study indicate that psychological demands, decision latitude, and support from fellow students or teachers can be described in a valid and reliable manner in a university study setting, by a modified version of the DCS-instrument which has been extensively used and in research on psychosocial factors and health in the general workforce.

## Paper context

Universities are frequently treated as workplaces for students although they also represent a unique context. Existing research shows the importance of occupational stress on a range of physical and psychological outcomes. No valid instrument exists to measure this phenomenon among university students. This article validates a modified Demand-Control-Support instrument for student populations examining internal consistency and construct validity. The instrument produced can be used to examine psychological study environment for university students in different contexts.
